# Surgical Removal of The Mechanical Valve Leaflet Dislocated into The Pulmonary Vein

**DOI:** 10.21470/1678-9741-2021-0350

**Published:** 2022

**Authors:** Djordje Zdravkovic, Igor Zivkovic, Vladimir Kovacevic, Petar Milacic, Miroslav Milicic

**Affiliations:** 1 Department of Cardiac Surgery, Dedinje Cardiovascular Institute, Belgrade, Serbia.; 2 Department of Radiology, Dedinje Cardiovascular Institute, Belgrade, Serbia.; 3 School of Medicine, University of Belgrade, Belgrade, Serbia.

**Keywords:** Aortic Valve, Pulmonary Veins, Tomography, X-Ray Computed, Mitral Valve

## Abstract

Accidental detachment of mechanical valve leaflet during implantation is a rare and potentially serious complication. When the lost leaflet cannot be found by direct visualisation, additional diagnostic procedures are necessary to detect it. Computer tomography is the best detection method, but the patient needs reoperation. We presented a patient in whom the detached leaflet migrated and became trapped into the left inferior pulmonary vein. The computed tomography (CT) scan was used to reveal leaflets, and successful extirpation was performed in the second operation.

**Table:** Abbreviations, Acronyms & Symbols

CT	= Computed tomography

## INTRODUCTION

Accidental detachment of pyrolytic carbon hemidisc from the artificial valve is a rare and serious complication of the surgical aortic valve replacement procedure. It is most frequently caused by inadequate surgical manipulation or catheterization procedures in patients with a previously implanted mechanical valve^[1-3]^. The literature described spontaneous delayed leaflet detachment and embolization due to a structural defect of the artificial valve^[4]^. If a fracture occurs intraoperatively, part of the valve can generally be retained into the heart cavities or migrate distally into the thoracoabdominal aorta or aortic bifurcation. When the disc is fragmented, it can be found in the iliac, femoral or even popliteal arteries^[4,5]^. Experience shows that such an escaped leaflet is difficult to detect. If it is not possible to find the leaflet by direct visualization, additional diagnostic procedures can be used. Historically, computed tomography (CT) scan has been more successful than plain radiography, angiography, and ultrasound^[1]^.

### Case Presentation

A 57-year old male was admitted to the hospital due to surgical aortic valve replacement. The procedure was performed through a partial upper ministernotomy. Cardiopulmonary bypass was instituted in a standard fashion. The vent was placed through the right superior pulmonary vein into the left atrium. The ascending aorta was clamped, and the heart was arrested by a blood cardioplegic solution. Transverse aortotomy was performed 1.5 cm above the origin of the right coronary artery. Excision of the severely calcified valve was performed with extensive decalcification of the annulus. The 21-mm mechanical aortic valve prothesis St Jude Medical Regent was sewn using simple pledgetted stitches and lowered to the aortic annulus using the original holder. During the tying of the sutures at the level of the commissure between right and noncoronary cusps, it was noticed that the right hemidisc of the prosthesis was missing. The artificial valve was immediately explanted from the position. Inspection of the left ventricle was made through the aorta, but the hemidisc was not found. Conversion to full sternotomy was done, and the left atrium was opened through the Sondergaard's groove. The left ventricle and the left atrium were visually and manually inspected without results. To better visualize the cardiac chamber, the endoscopic camera was brought through the aortic annulus into the left ventricle, but the detached hemidisc could not be identified. Due to the prolonged duration of cardiopulmonary bypass, it was decided to continue with the operation. Another mechanical prosthesis, St Jude Regent valve size 21 mm, has been implanted. After restarting heart contractions, intraoperative transesophageal examination was unsuccessful in detecting the missing leaflet.

**Fig. 1:**
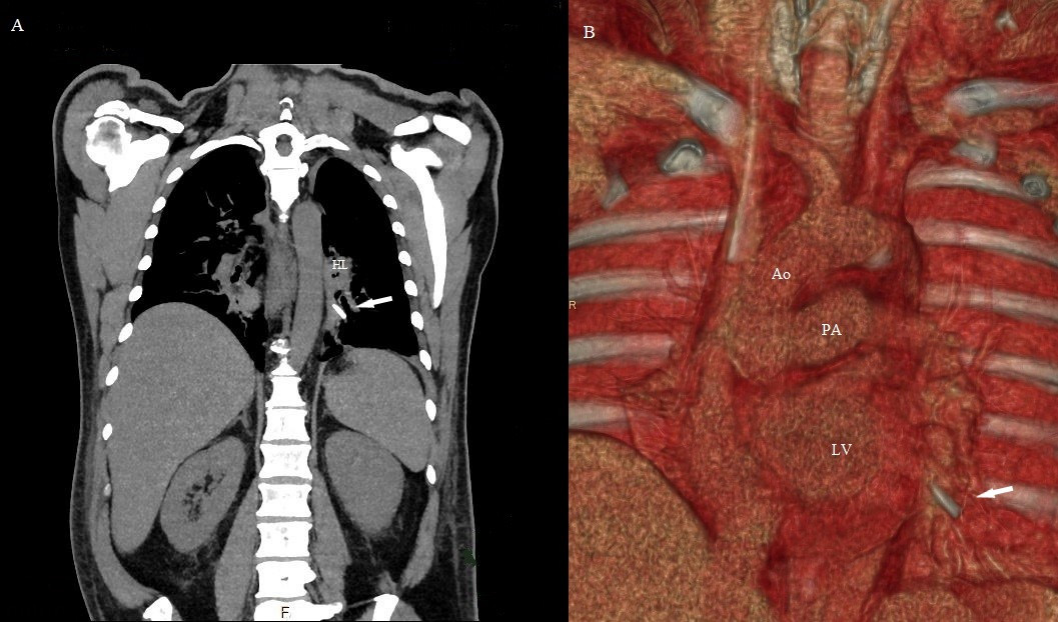
CT scan revealed a mechanical aortic valve hemidisc into the inferior left pulmonary vein. (A) Native computed tomography scan. (B) 3D volume rendering reconstruction of the chest. White arrow=artificial valve hemidisc. Ao=aorta; HL=hilum of the lung; LV=left ventricle; PA=pulmonary artery

**Fig. 2:**
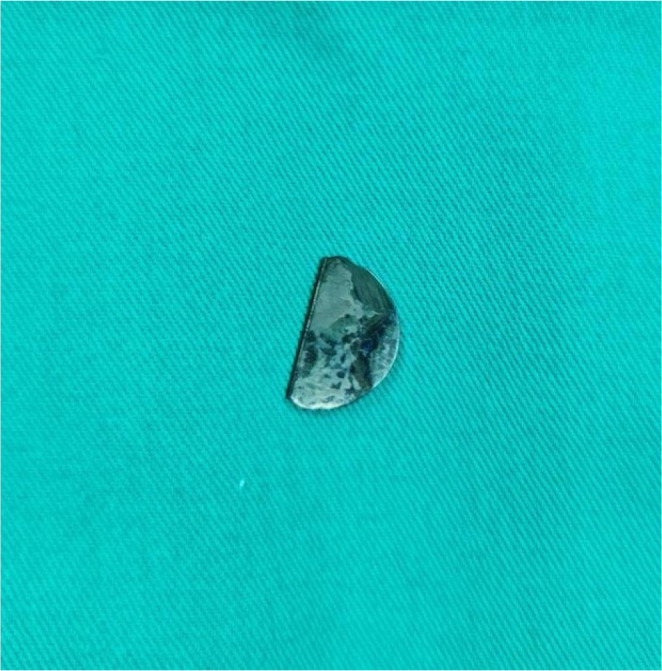
Extracted mechanical valve hemidisc.

**Fig. 3:**
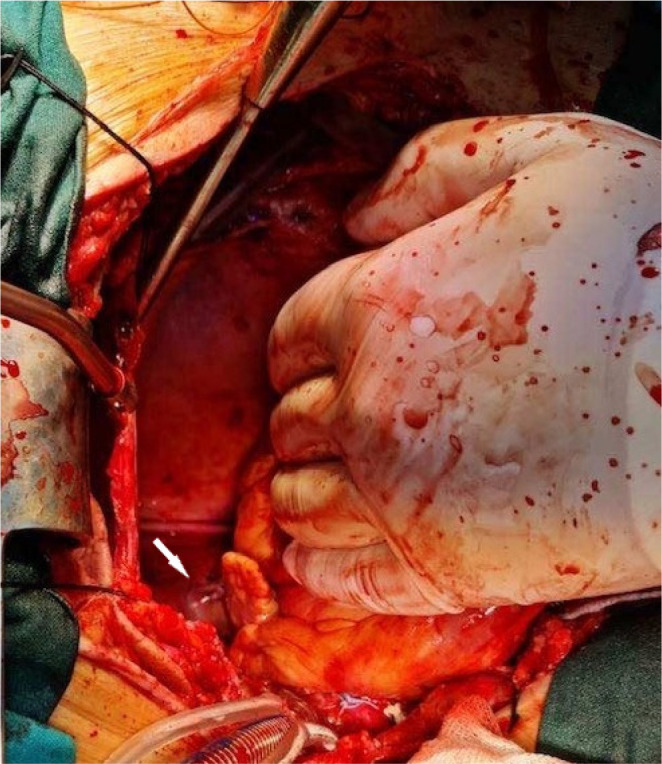
Surgical view of the sutured left inferior pulmonary vein. White arrow = left inferior pulmonary vein

The patient was weaned from bypass and referred to the intensive care unit in a stable condition. Extubation was performed seven hours after the procedure. Chest radiography and transthoracic echocardiography did not identify a foreign body in the thorax. Subsequently, a CT chest scan was performed, and the missing leaflet was visualized about 2.5 cm deep into the left inferior pulmonary vein (Figures 1A and B). The patient was immediately transferred to the operating room. Reoperation was performed through median sternotomy using standard cardiopulmonary bypass. The aorta was clamped, and the heart was arrested with blood cardioplegia. Two deep stay stitches were placed on the left side of the pericardium to lift the heart into a good position and satisfactory visualization of left pulmonary veins and atrium. A 1.5 cm longitudinal incision was made just before the bifurcation of the tree, and a hemidisc was detected trapped at the level of the 1^st^ bifurcation of the left inferior pulmonary vein. The valve part was completely pulled out using forceps (Figure 2). Deaeration and suturing of the vein were performed (Figure 3). The patient was successfully weaned from bypass. The postoperative course was uneventful, and the patient was discharged from the hospital on the 6^th^ postoperative day.

## COMMENT

Only a few cases of leaflets accidentally detached from the mechanical valve, which were retained in the heart chambers, were reported. Raut et al.^[6]^ described a case in which a missing leaflet of the mechanical aortic valve was found in the left atrium. Eldreth et al.^[1]^ reported an escaped fragment of the aortic prosthesis detected in an atherosclerotic plaque in the abdominal aorta two years after implantation. They also stated about 30 cases of escaped leaflet published by various authors, of which 17 were successfully found and removed with a mean extraction time of 21 days.

The location of the escaped leaflet (in our case, in the left inferior pulmonary vein) is unexpected. To the best of our knowledge, this is the first case in which part of the mechanical valve has become detached, dislodged and trapped into the left pulmonary vein. It is difficult to explain how the leaflet from the aortic position primarily passed the mitral valve and entered the left inferior pulmonary vein. Most likely, migration of the detached leaflet occurred before the heart began to contract. Considering that neither chest radiography nor echocardiography exam did not identify a foreign body, an early postoperative CT scan is probably the only way to visualize detached hemidiscs. Pyrolytic carbon, the material the leaflet is made of, is invisible to radiography and ultrasound.

## CONCLUSION

Intracardiac detachment and loss of the mechanical valve leaflet is an infrequent but serious complication of surgical valve replacement. For this intraoperative complication, detailed examinations of the heart chambers are obligatory to find the missing leaflets. Unfortunately, in case of detection failure, a CT scan of the chest or the whole body is necessary to identify the foreign body. Early reoperation is mandatory to evacuate the missing leaflet and prevent adverse events.

**Table:** Authors’ Roles & Responsibilities

DZ	Substantial contributions to the conception or design of the work; or the acquisition, analysis, or interpretation of data for the work; drafting the work or revising it critically for important intellectual content; agreement to be accountable for all aspects of the work in ensuring that questions related to the accuracy or integrity of any part of the work are appropriately investigated and resolved; final approval of the version to be published
IZ	Substantial contributions to the conception or design of the work; or the acquisition, analysis, or interpretation of data for the work; drafting the work or revising it critically for important intellectual content; agreement to be accountable for all aspects of the work in ensuring that questions related to the accuracy or integrity of any part of the work are appropriately investigated and resolved; final approval of the version to be published
VK	Substantial contributions to the conception or design of the work; or the acquisition, analysis, or interpretation of data for the work; drafting the work or revising it critically for important intellectual content; agreement to be accountable for all aspects of the work in ensuring that questions related to the accuracy or integrity of any part of the work are appropriately investigated and resolved; final approval of the version to be published
PM	Substantial contributions to the conception or design of the work; or the acquisition, analysis, or interpretation of data for the work; drafting the work or revising it critically for important intellectual content; agreement to be accountable for all aspects of the work in ensuring that questions related to the accuracy or integrity of any part of the work are appropriately investigated and resolved; final approval of the version to be published
MM	Substantial contributions to the conception or design of the work; or the acquisition, analysis, or interpretation of data for the work; drafting the work or revising it critically for important intellectual content; agreement to be accountable for all aspects of the work in ensuring that questions related to the accuracy or integrity of any part of the work are appropriately investigated and resolved; final approval of the version to be published
